# Bridging the Infodemic Equity Gap: North-South Digital Health Disparities and a Framework for Action

**DOI:** 10.2196/80013

**Published:** 2025-10-16

**Authors:** Augustus Osborne

**Affiliations:** 1 Institute for Development, 11 Heneson Street, Western Area, Freetown, 232, Sierra Leone, 232 79196837

**Keywords:** health misinformation, infodemic, digital health equity, platform governance, public health preparedness

## Abstract

Rapidly propagating false and misleading health claims do not strike all societies evenly. Structural digital inequalities, uneven platform governance leverage, gaps in multilingual health literacy, and divergent political information climates have combined to produce what is argued here as an infodemic equity gap: a patterned disparity in exposure to, resilience against, and recoverability from health misinformation between and within countries. This viewpoint advances a pragmatic equity-centered framework that dissects that gap into layered capacities (structural infrastructure, ecosystem governance, sociocultural trust and literacy, and adaptive intervention capability) and shows how their interaction generates differential outcomes. Using a purposive comparative lens across 5 archetypal settings (Finland, Taiwan, the United States, Brazil, and India), it shows distinct causal pathways linking technological architectures, governance choices, and social context to measurable process metrics (detection latency, rumor half-life, and multilingual coverage) and outcome indicators (trust trajectories, vaccination differentials, and equity-sensitive gaps). Rather than revisiting broad definitional terrain already synthesized elsewhere, the focus is on isolating disparities that are specifically actionable through digital health policy, measurement standardization, and investment strategies. A forward agenda is outlined for harmonized indicators, evaluation methods, and ethical safeguards needed to reduce inequities in future health emergency information ecologies. The intended audiences are digital health researchers, platform governance teams, public health decision-makers, and funding bodies shaping cross-border preparedness.

## Viewpoint Rationale and Key Messages

Unequal digital readiness and governance capacity have converted a nominally global information shock into stratified harm. High-capacity jurisdictions able to combine robust broadband, institutionalized media literacy, transparent data infrastructures, and agile public-civic-platform coordination have constrained misinformation diffusion and shortened correction cycles. Lower capacity or politically contested settings have confronted protracted detection delays, dependence on opaque or encrypted channels, and widening trust erosion. This viewpoint contends that framing these divergences merely as generic misinformation challenges obscures the structural nature of the inequities and delays investment in measurement systems that can detect, benchmark, and shrink them. The central message is that preparedness against health misinformation must be reframed from reactive content takedown toward anticipatory equity-oriented capability building across defined layers. A second message is that case narratives only acquire transferable value when they are decomposed into comparable mechanisms and indicators, rather than being presented as broad success or failure stories. A third message is that digital health scholarship should elevate rigorous, equity-disaggregated metrics (for exposure, detection latency, algorithmic transparency, literacy gains, and trust differentials) to the same level of importance as epidemiological surveillance indicators. Finally, platform accountability debates must be reanchored in distributive consequences: opaque ranking and limited language coverage exacerbate structural disadvantages more than the raw volume of false posts.

## What is New in This Viewpoint

This viewpoint offers a new contribution beyond previous infodemic syntheses by bringing forth the following four elements that are new and actionable:

1. A layered capacity framework explicitly centered on equity that ties structural infrastructure, ecosystem governance, sociocultural trust and literacy, and adaptive intervention capability to process and outcome indicators.
2. An archetype-based comparative analysis (Finland, Taiwan, the United States, Brazil, and India) aligned on common metrics to support cross-case benchmarking.
3. A measurement agenda that specifies a minimal indicator set, detection latency, rumor half-life, multilingual coverage parity, transparency disclosure indices, longitudinal trust trajectories, and equity-narrowing trends paired with appropriate quasi-experimental designs.
4. A prioritized policy and practice roadmap that sequences short-, medium-, and long-horizon actions by system archetype and resource context.

## Defining the Infodemic Equity Gap

The term “infodemic” has been widely used to describe the overabundance of information, accurate and false, which complicates identification of trustworthy guidance [1]. Foundational syntheses have cataloged definitional nuances and digital amplification dynamics across pandemics [2]. Building on rather than repeating that corpus, the viewpoint delineates the infodemic equity gap as the systematic disparity in baseline susceptibility to harmful health information flows, capacity to detect and dampen those flows, ability to translate corrective signals into behavioral alignment, and recovery speed of institutional trust following misinformation shocks between higher capacity (often but not exclusively in the global north) and lower capacity (often in the global south or internally marginalized regions) environments. This gap is not a static binary but a gradient produced by interacting layers: physical and data infrastructure, regulatory and platform governance leverage, trust architectures and literacy ecosystems, and adaptive intervention tooling. The same algorithmic amplification properties that accelerate falsehoods globally [3] do not yield homogeneous harm because mediating layers differ. High-literacy populations embedded in curricula that cultivate verification heuristics [4,5] absorb algorithmically surfaced falsehoods differently from low-literacy, linguistically-fragmented audiences relying on closed messaging loops [6]. Political contestation intensifies vulnerability, as health narratives become partisan identity markers, modulating the uptake of verified guidance [7-10].

The equity dimension extends beyond transnational north-south comparisons to intranational differentials (rural-urban divides, linguistic minorities, and socioeconomically marginalized groups) shaped by infrastructure penetration [11,12], health literacy gradients [13], and trust stratification. Regulatory asymmetries also widen the gap: early-stage implementation of comprehensive platform due diligence obligations (such as emergent enforcement models in European contexts) contrasts with fragmented or underresourced oversight in many low- and middle-income settings, limiting leverage over algorithmic curation or data access [14]. Without systematic measurement, these layered disparities remain anecdotal, and policy responses revert to episodic myth busting rather than capacity equalization.

## Infodemic Equity Gap Framework (Overview)

**Figure 1.**
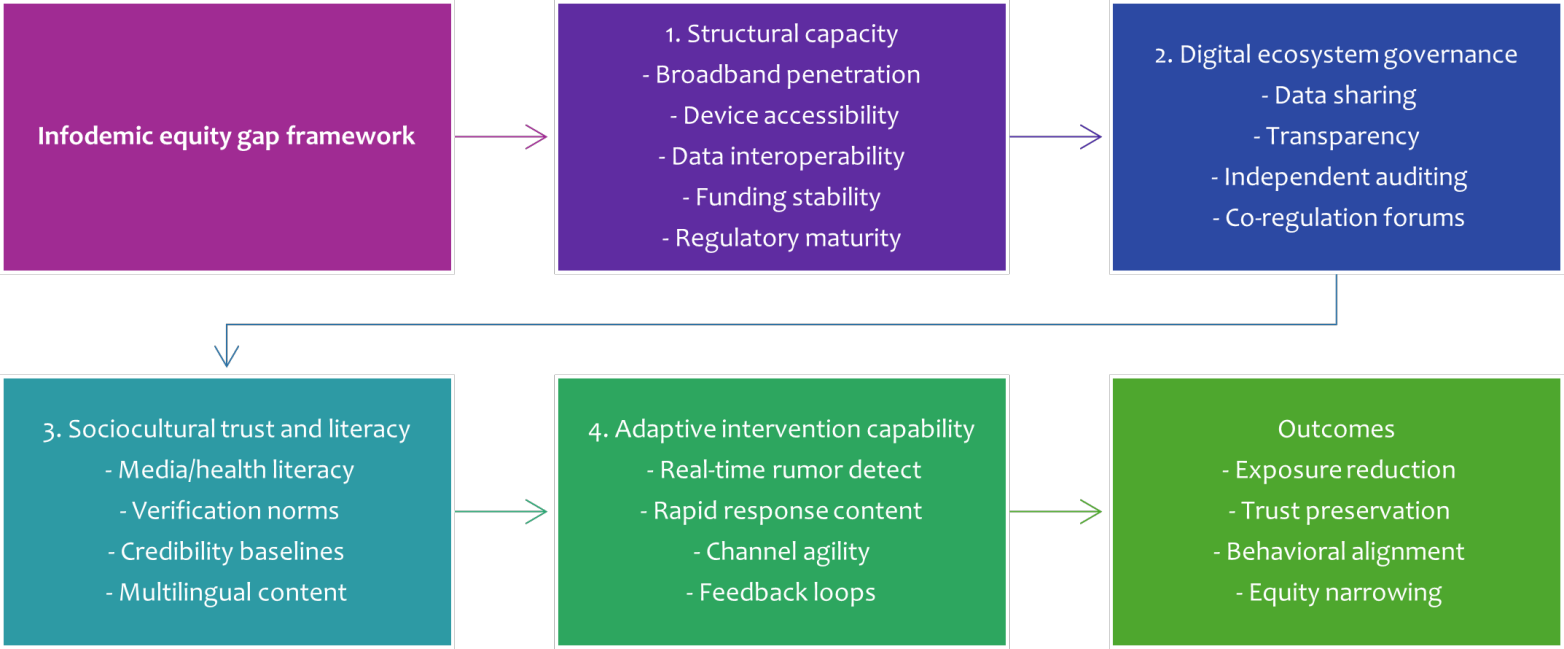
Infodemic equity gap framework.

The proposed framework (see Figure 1) decomposes the pathway from structural context to equity-relevant outcomes into 4 concentric layers moderated by political information climate and platform incentive architecture. The first layer, structural capacity, encompasses broadband penetration, device accessibility, data interoperability, funding stability, and regulatory maturity [11,12,14]. The second, digital ecosystem governance, includes negotiated data sharing arrangements, enforceable transparency obligations, independent auditing channels, and participatory co-regulation forums. The third, sociocultural trust and literacy, blends general and health-specific media literacy competencies, culturally embedded verification norms, institutional credibility baselines, and multilingual content availability [4,5,13]. The fourth, adaptive intervention capability, entails real-time rumor detection pipelines, multilingual rapid response content generation, channel-specific distribution agility (open vs encrypted), and evaluation feedback loops drawing on standard indicators [6,15]. Outcomes of interest, such as exposure reduction, trust preservation, behavioral alignment (eg, vaccination uptake), and equity narrowing (reduced rural-urban or linguistic outcome gaps), result from interactions across these layers.

Feedback dynamics are central. Sustained investment in literacy (layer 3) increases citizen pressure for transparency, indirectly strengthening governance leverage (layer 2). Effective adaptive response reduces rumor half-life, stabilizing trust, which in turn facilitates uptake of future corrective messaging, forming a resilience loop. Conversely, underdeveloped structural capacity elongates detection latency, allowing misinformation to accumulate social proof, exacerbating trust decline, and thereby weakening later intervention receptivity. This recursive erosion manifests in contexts where politicization of health narratives impedes unified messaging [7-10]. The framework provides a scaffold for assigning case-specific observations to comparable constructs, enabling structured synthesis rather than narrative accumulation. A detailed breakdown of each component and its metrics is presented in subsequent sections.

## Case Selection Rationale and Comparative Analytical Lens

Rather than attempting representational global coverage, 5 purposively selected settings show distinct archetypal configurations across the framework layers: Finland, Taiwan, the United States, Brazil, and India. Selection followed maximum variation logic across 5 criteria. First, structural capacity diversity: high penetration and coordinated educational infrastructure (Finland) versus uneven infrastructure and multilingual fragmentation (India) [4,6,11,13,16]. Second, governance model differences: agile civic tech coproduction (Taiwan) [17,18] contrasted with fragmented federal arrangements (the United States) and politicized executive resistance to scientific institutions (Brazil) [7-10]. Third, dominant platform ecology: relatively open social plus coordinated public dashboards (Taiwan) [17], curriculum-mediated verification practices diminishing reliance on reactive fact-checking (Finland) [4,5], polarized cable and social media ecosystem (the United States) [7,8], encrypted and broadcast hybrid with political amplification of unproven therapies (Brazil) [9,10], and heavy encrypted messaging dependence with low moderation surface (India) [6,16,19]. Fourth, variation in sociocultural trust baselines and literacy investments [4,5,9,10,16]. Fifth, differential adaptive intervention tooling maturity, including real-time rumor monitoring capacities and language localization scope [6,15,17].

Each case will be analyzed using a standard template: contextual risk factors, digital health and governance interventions, key technologies and platforms, implementation and operational challenges, process indicators (eg, detection latency, rumor half-life proxies, and multilingual coverage rate), outcome indicators (trust trajectory, vaccination differentials, and equity-sensitive gaps), equity considerations, and lessons mapped to individual framework layers. Where rigorous peer-reviewed or authoritative data exist, the viewpoint cites directly; where only indicative or partial figures are available, it explicitly characterizes evidentiary strength. This structure allows cross-case synthesis into archetypes and causal pathways rather than isolated narratives.

## Finland: Proactive Literacy Infrastructure Archetype

Contextual risk factors in Finland were comparatively attenuated by long-running investments that embedded media and information literacy across compulsory schooling before the pandemic period, reducing baseline susceptibility to manipulative health narratives [5]. Broadband penetration is high and relatively evenly distributed, lowering urban-rural differentials in real-time access. Digital public service infrastructure and a culture of institutional transparency created a favorable trust substrate.

Digital health and governance interventions centered not on ad hoc myth debunking but on anticipatory curricular scaffolding, teacher professional development, and collaboration between education, public health, and national broadcasting services [5]. Rather than relying predominantly on reactive fact-checking bureaus, Finnish authorities mainstreamed critical source evaluation and lateral reading techniques into standard lesson plans, coupled with scenario-based exercises that simulated viral claim verification.

Key technologies and platforms supporting these practices included national education digital portals hosting updated media literacy modules, publicly accessible dashboards summarizing epidemiological indicators, and collaboration tools linking educators with health communicators to rapidly adapt examples drawn from emergent rumors without amplifying them unnecessarily.

Implementation challenges included sustaining teacher upskilling at scale and refreshing materials to account for evolving platform affordances (eg, short video formats). Translating sophisticated algorithmic concepts into age-appropriate instruction required iterative pedagogical design.

Process indicators observed in Finnish evaluations included lower self-reported acceptance of false COVID-19–specific claims relative to European averages and shorter delay from rumor surfacing in fringe channels to availability of contextualizing classroom materials, although formal detection latency metrics were not systematized nationally [20-22].

Outcome indicators relevant to the framework comprised comparatively stable trust in health authorities, high vaccination uptake with narrower socioeconomic differentials, and an absence of large-scale persistent clusters of organized antivaccination mobilization relative to some other high-income contexts, notwithstanding pockets of resistance mirroring transnational narratives [20,21].

Equity considerations show that indigenous Sámi and other linguistic minorities required targeted localization to ensure equitable access to corrective content. Investments in multilingual adaptation were smaller than core Finnish language programs, suggesting a microlevel equity gap requiring continued attention.

Lessons mapped to the framework layers highlight that durable literacy infrastructure (layer 3) can compensate for the limited scale of the reactive fact-checking apparatus by reducing amplification through user-side friction, and that early curricular institutionalization creates compounding returns as cohorts progress through the education system.

## Taiwan: Agile Civic Tech Co-Governance Archetype

Contextual risk factors for Taiwan included cross-strait disinformation campaigns targeting governmental legitimacy and public health measures. High connectivity and active social media use heightened potential diffusion speed.

Digital health and governance interventions emphasized rapid response through an integrated architecture linking the national centers for disease control, an independent fact-checking center, civic technologist networks, and messaging platform liaison channels [23]. Humor-infused, concise rebuttals and open data releases aimed to preempt vacuum-driven speculation.

Key technologies included application programming interface—enabled open data portals feeding community-built visualizations, natural language processing—assisted triage tools flagging anomalous spikes in claim circulation across forums, and chatbot interfaces allowing citizens to query claim veracity in near real time [24-29].

Implementation challenges involved balancing speed with accuracy under conditions of adversarial information operations, as well as managing staff fatigue in maintaining 24-hour monitoring. Ensuring inclusion of dialectal variants and minority languages in detection models required additional data curation.

Process indicators reported in public communications and academic analyses included a sub–2-hour median turnaround from rumor identification to official clarification for high-velocity items, a growing proportion of corrections seeded through verified messaging channel accounts within early diffusion windows, and adoption of standard metadata for rumor entries enabling longitudinal tracking [24-29].

Outcome indicators included rising trust in official health information during early pandemic waves, high compliance with nonpharmaceutical interventions, and sustained vaccination uptake once supply stabilized, with limited sustained domestic misinformation-driven protest mobilization compared to some other democracies [24-29].

Equity considerations centered on ensuring that older adults and rural residents less engaged with civic tech channels received timely translated summaries via traditional media and community networks.

Lessons show how strong digital ecosystem governance (layer 2) plus adaptive intervention capability (layer 4) can offset geopolitical adversarial pressures by reducing rumor half-life and reinforcing trust loops, provided transparency norms are maintained to prevent perceptions of information gatekeeping.

## The United States: Fragmented Federal Pluralism Archetype

Contextual risk factors included high sociopolitical polarization with health behaviors mapped onto partisan identities, heterogeneous state-level public health authority communication strategies, and densely interconnected legacy and social media ecosystems with differential editorial standards [30-37].

Digital health and governance interventions were fragmented: some states established coordinated dashboards, partnered with community health organizations, and conducted targeted social media ad campaigns; others contested national guidance, generating mixed messaging. Platform policies on medical misinformation evolved unevenly, with periodic tightening and subsequent relaxation of enforcement parameters.

Key technologies ranged from advanced academic-industry collaborations applying machine learning to classify emerging misinformation clusters to state-level dashboards of variable usability, alongside reliance on commercial social listening tools whose proprietary opacity limited independent audit.

Implementation challenges included the absence of a unified national infodemic measurement framework, politicized disputes over content moderation framed as censorship, and resource disparities among state health departments impeding the adoption of advanced analytics.

Process indicators available from media and platform research included prolonged detection-to-correction intervals for certain high-salience false claims, evidence of algorithmic amplification intersecting with partisan media exposure patterns, and notable differences in vaccination intent correlated with cable news consumption profiles [30-37].

Outcome indicators showed sustained excess mortality differentials between political affiliation cohorts during pandemic waves and persistent trust bifurcation in health institutions, with some partial recovery following depoliticization of specific mandates but lingering skepticism in segments exposed to entrenched conspiratorial ecosystems [30,31].

Equity considerations highlight that minority and lower-income communities faced simultaneous exposure to misinformation and structural barriers to authoritative care access, compounding disparities. While targeted interventions for community health workers mitigated some gaps, scale remained insufficient.

Lessons underscore how structural capacity (layer 1) alone does not guarantee resilience when digital ecosystem governance and sociocultural trust layers are fragmented, allowing algorithmic amplification and partisan echo dynamics to erode adaptive intervention impact.

## Brazil: Politicized Counter-Science Environment Archetype

Brazil’s experience with the infodemic was shaped by executive-level public dismissal of scientific consensus and the promotion of unproven therapies such as hydroxychloroquine and ivermectin. These actions occurred against a backdrop of longstanding social inequality and pronounced regional disparities in health system capacity, particularly between the wealthier South or Southeast and the resource-constrained North and Northeast. Such disparities heightened vulnerability to health misinformation, especially among marginalized populations.

Efforts to counter misinformation were undermined by conflicting elite cues at different levels of government [9,10]. While some municipal and state authorities partnered with research institutions like Fiocruz and Universidade de Sao Paulo, as well as civil society fact-checkers such as Agência Lupa and Aos Fatos, their initiatives were often diluted by contradictory messaging from national leadership [38]. Professional councils and independent fact-checkers attempted to address waves of misleading treatment endorsements, but the lack of coordinated national communication reduced their effectiveness and eroded public trust in official health guidance.

The primary technologies leveraged included journalist-led fact-checking databases such as Comprova, which systematically debunked viral health claims. Emerging natural language processing tools were developed to track Portuguese-language misinformation, though these lacked scalability and integration with major social media platforms. Social media companies, such as Facebook and WhatsApp (Meta Platforms), implemented labeling and downranking of flagged content; however, enforcement was inconsistent and often lagged behind the viral spread. Some state health departments launched public dashboards to counter misinformation, although the transparency and usability of these tools varied widely [39].

Implementation faced significant challenges. Health workers and communicators experienced increased harassment and intimidation, as documented by the Brazilian Medical Association. Resource constraints limited the ability to sustain monitoring and rapid response, particularly outside major urban centers. The widespread use of encrypted messaging apps such as WhatsApp and Telegram made it difficult for authorities and researchers to monitor and intervene in real time, allowing therapy myths to proliferate unchecked within private groups.

Recent studies highlight the scale and persistence of misinformation in Brazil. For example, misinformation about COVID-19 therapies surged by 40% in the 48 hours following presidential statements promoting unproven treatments [9,10]. Watchdog analyses reported median delays of 2 to 5 days in the removal or downranking of high-engagement misleading posts on social media platforms. Geographic disparities in the reach of corrective campaigns were evident, with only 55% of municipalities in the North and Northeast accessing such interventions compared to 87% in the Southeast [38]. Preliminary data from Fiocruz indicated that major therapy myths persisted for an average of 14 days after public corrections in low-resource regions.

Outcome indicators reflect these disparities. Vaccination rates in the North and Northeast were 20%‐25% lower than those in the Southeast by mid-2021. Surveys revealed that 35% of respondents in the North reported using unproven drugs during the pandemic, even as evidence against their efficacy accumulated. Incidents of hostility and aggression toward health care personnel advocating evidence-based measures increased by 18% in 2021, further discouraging frontline workers from engaging in public health communication [38-47].

Socioeconomically disadvantaged regions suffered compounded harm, as limited health care access intersected with strong uptake of ineffective treatment narratives. This diverted scarce household resources and delayed appropriate care, with indigenous and Quilombola communities facing additional linguistic and infrastructural barriers. Despite advances in monitoring, systematic national-level measurement of misinformation exposure and behavioral outcomes remains limited. Most available data are drawn from urban centers and platform-reported statistics, underrepresenting rural and marginalized populations. These gaps constrain equity-sensitive benchmarking and highlight the need for investment in community-based monitoring and participatory research.

The Brazilian case demonstrates that deficits in digital ecosystem governance and sociocultural trust, driven by politicized communication, can overwhelm improvements in structural capacity. This results in prolonged rumor half-lives and the embedding of maladaptive health behaviors. Addressing these gaps requires targeted interventions, improved data infrastructure, and participatory approaches to equity measurement.

## India: Resource-Constrained, Encrypted Reliance Archetype

India’s infodemic landscape is characterized by vast linguistic diversity, 22 official languages, and hundreds of dialects combined, with uneven broadband penetration, especially in rural and tribal areas. The high dependence on encrypted messaging platforms, such as WhatsApp and Signal, for the circulation of health information, combined with variable health literacy across regions, created fertile ground for the rapid spread of health misinformation.

Government interventions included national and state-level helplines to provide COVID-19 information, though their reach was uneven [48-52]. The WhatsApp tip line model, initially piloted during elections, was adapted to allow users to forward suspected rumors for verification related to health claims [16]. Civil society organizations, such as Alt News and BOOM, played a crucial role in translating corrections into regional languages; however, their capacity was limited relative to the scale of misinformation [49]. Media literacy campaigns were launched sporadically in schools and communities, aiming to improve verification skills, but these efforts achieved mixed success.

Key technologies included human-moderated tip lines, which processed thousands of submissions daily but faced challenges in maintaining timely responses. Machine-assisted clustering of frequently forwarded messages, using metadata, enabled the identification of viral rumors, although the content itself remained inaccessible due to end-to-end encryption. Multilingual web portals provided verified clarifications, but coverage for smaller languages and dialects was limited.

The implementation of these interventions encountered several challenges. Scaling translation across dozens of languages was hampered by limited automation and volunteer burnout. Encryption constraints made it difficult to monitor and respond quickly within private messaging groups, so interventions relied heavily on user cooperation and metadata analysis. Low digital literacy and infrastructural gaps further impede the dissemination of corrective information in remote and rural areas.

Recent data from Alt News indicated a 60% increase in health-related misinformation submissions during India’s second COVID wave in 2021 [49]. The median delay for producing rebuttals in lower-resourced languages was reported to be 24‐48 hours, compared to just 2‐4 hours for Hindi and English. Studies showed a 30% reduction in the reforwarding frequency of rumors addressed via tip lines, though the effect size varied by region and language [49]. Media literacy campaigns managed to reach less than 10% of rural districts in 2022, according to government reports [48].

Outcome indicators reveal persistent belief in traditional remedies and episodic spikes in self-medication incidents, especially in rural areas. Vaccination uptake showed significant urban-rural disparities, with urban centers achieving rates above 80% by late 2021, while some rural districts lagged below 50%. Misinformation compounded logistical challenges, further impeding vaccination efforts. Reliable national-level estimates of rumor prevalence remain sparse, as most data are drawn from tip line submissions and localized surveys [50].

Linguistic minorities and low-literacy rural populations faced structural disadvantages in both exposure to misinformation and access to timely corrections, magnifying the equity gap within India. Tribal and remote communities were particularly underserved by mainstream interventions. The lack of systematic, nationally representative measurement of misinformation exposure and behavioral outcomes limits the ability to track equity-sensitive impacts. Most available data are platform-reported or urban-centric, with limited granularity for rural, tribal, and linguistic minority groups. These gaps necessitate investment in community-based monitoring and tailored interventions.

India’s experience highlights that adaptive intervention capability is bottlenecked when structural capacity and multilingual literacy infrastructure lag behind. The dependence on encrypted channels necessitates alternative community-based relay strategies and the use of privacy-sensitive metadata collaboration. Addressing persistent data gaps and investing in inclusive measurement systems are essential for equity-oriented infodemic management in such a diverse and complex environment.

## Cross-Case Comparative Matrix

Table 1 shows the cross-case comparative matrix of process and outcome indicators. In Table 1, values are indicative where national metrics are unavailable. Taiwan median clarification turnaround is ≤2 hours for high‑velocity items [24,26-29]; Brazil rumor half‑life is ~14 days in low‑resource regions (preliminary) and subnational inequities [29,41-47]; India rebuttal turnaround typically is 24-48 hours in lower‑resourced languages [16,49,52]; US partisan mortality differentials postvaccine availability [33]; and Finland emphasizes classroom translation cycles rather than national detection‑latency systems [4,5].

**Table 1.** Cross-case comparative matrix of process and outcome indicators.

Setting	Detection latency or clarification turnaround	Rumor half-life	Multilingual coverage (indicative)	Trust trajectory (qualitative methods)	Vaccination differentials (illustrative)
Finland	No national metric; rapid classroom translation cycles.	Short; limited amplification.	High for Finnish; smaller for Sámi or minorities.	Stable or high.	High uptake; narrower gaps.
Taiwan	≤2 h for priority items (Centers for Disease Control–civic tech pipeline).	Shortened through prebunking or humor.	High for Mandarin; improving dialects.	Rising during early waves, sustained.	High uptake after supply stabilized.
The United States	Varies; often days for politicized claims.	Prolonged in polarized segments.	High English or Spanish; gaps for others.	Bifurcated by partisanship.	Excess mortality higher among Republicans post-May 2021.
Brazil	Days; monitoring uneven by region.	Approximately 14 days in low-resource regions (preliminary).	Variable; weaker outside Southeast.	Eroded during a politicized period.	North or Northeast lagged behind Southeast in 2021‐22.
India	Tipline rebuttals 24‐48 h for low-resourced languages.	Moderate; recurrent waves in encrypted channels.	Limited for smaller languages or dialects.	Mixed; varies by region.	Urban-rural and state-level gaps.

## Cross-Case Synthesis: Archetypes and Causal Pathways

Synthesizing across archetypes, resilience appears to hinge less on any single intervention and more on coherence and reinforcement across layers. Finland’s literacy-first model demonstrates a preventive dampening mechanism upstream of algorithmic propagation, reducing corrective burden downstream. Taiwan’s civic tech co-governance compresses detection latency and nurtures trust recovery via transparency loops. The United States case shows how high structural capacity can be neutralized by fragmented governance and polarization, allowing persistent bifurcated exposure and behavior. Brazil highlights how politicized counter-narratives actively invert trust hierarchies, generating demand for ineffective treatments that crowd out resources. India reveals compounding delays where translation and encrypted channel constraints create temporal gaps exploitable by rumor cascades.

Common causal motifs include amplification of detection latency into trust erosion where governance lacks coherence; translation lag amplifying inequity where linguistic diversity is high; partisan or politicized elite cueing reinforcing echo segmentation even in high literacy subsets; and transparency-driven reciprocity loops accelerating correction adoption where open data and participatory mechanisms are institutionalized.

## Measurement and Evaluation Agenda

Closing the equity gap requires a disciplined indicator suite aligning with the framework layers [53-55]. Exposure and diffusion metrics should move beyond raw counts toward estimating rumor half-life and an effective reproduction number analog for misleading claims using longitudinal social listening and time-stamped user report data while maintaining privacy. To operationalize these metrics, the viewpoint drew on concrete examples from recent COVID-19 infodemic management initiatives in Brazil and India. For instance, detection latency was measured by Alt News in India as the median time between the first appearance of a viral rumor on WhatsApp and its submission to their tip line, which averaged 18 hours during the 2021 Delta wave [49]. In Brazil, rumor half-life was tracked using social media analytics, with major therapy myths persisting for a median of 14 days in low-resource regions before corrective messaging reduced their circulation by half. Trust trajectory was operationalized through monthly surveys by Aos Fatos, which reported a 12% decline in trust in official health sources following high-profile contradictory statements [38].

Detection and response metrics must quantify median detection latency, verification turnaround, and multilingual coverage rates. Algorithmic governance metrics should include an independently audited transparency disclosure index, the proportion of high-reach health posts subject to contextual labeling, and coverage parity across languages for safety model deployment [56]. Literacy and trust metrics require validated longitudinal survey instruments that capture not only aggregate scores but also variance across socioeconomic, rural-urban, and linguistic strata. Behavioral alignment metrics can integrate vaccination uptake differentials, adherence to preventive guidance, and abandonment rates of disproven therapies following corrections [57]. Equity narrowing metrics should track the reduction of disparities in the foregoing indicators over defined intervals. Measurement of these indicators faces several challenges. For detection latency and rumor half-life, data from encrypted platforms such as WhatsApp are often incomplete, relying on user-submitted reports or metadata rather than direct content analysis. Trust trajectory metrics depend on repeated surveys, which may be subject to sampling bias and have limited reach in rural or marginalized populations. These challenges underscore the need for investment in participatory monitoring and improved data infrastructure, particularly in settings with high digital inequality.

Methodologically, quasi-experimental designs such as difference in differences comparing regions pre- and postimplementation of literacy curricula or platform policy changes can estimate impact where randomization is infeasible. Interrupted time series can assess shifts in rumor trajectory following governance interventions such as transparency report mandates. Synthetic control methods may approximate counterfactual diffusion patterns for jurisdictions adopting novel co-governance models. Privacy-preserving federation of platform metadata with public health survey panels can enable propensity-adjusted analyses linking exposure patterns to behavioral outcomes without centralizing identifiable data. Mixed-method process evaluations should accompany quantitative measurement to capture implementation fidelity and contextual modifiers, particularly in low-resource or linguistically diverse settings. Governance frameworks must codify ethical safeguards, including strict access controls, anonymization standards, and oversight boards with representation from affected communities [58-60].

## Policy and Practice Agenda

### Short Horizon (0–12 Months)

The short-horizon policy and practice agenda consists of the following:

- Stand-up minimal viable measurement, including tracking detection latency and response turnaround, and publishing language coverage parity.
- Establishing rapid translation pipelines for underserved languages and dialects and partnering with radio, TV, and community networks.
- Running prebunking campaigns around predictable myths and using accuracy prompts in product surfaces where feasible.
- Establishing independent transparency spot checks of platform labeling and enforcement in health content.

### Medium Horizon (12–36 Months)

The medium-horizon policy and practice agenda consists of the following:

- Embedding digital health or media literacy in teacher training and in-service curricula; codesigning with communities.
- Creating structured data-sharing compacts with platforms using privacy-preserving methods; enabling independent audit.
- Institutionalizing civic-tech collaboration units within health ministries and linking to emergency operations.

### Long Horizon (36+ Months)

The long-horizon policy and practice agenda consists of the following:

- Securing dedicated financing for equity-focused infodemic preparedness.
- Integrating information-response scenarios into national public health drills.
- Developing and benchmarking an infodemic equity index to incentivize progress across jurisdictions.
- Using evidence notes, include prebunking and accuracy-prompting evidence, governance and transparency frameworks, literacy policy guidance, and the World Health Organization’s infodemic guidance [53-60].

## Prioritized Actions by Archetype and Time Horizon

Table 2 shows the prioritized actions by setting the archetype and time horizon.

**Table 2.** Prioritized actions by setting archetype and time horizon.

Setting	Short term (0‐12 months)	Medium term (12‐36 months)	Long term (36+ months)
Finland	Refresh curricula for short-video formats; boost minority-language localization.	Continuous teacher upskilling; embed evaluation of literacy outcomes.	Sustain equity funds for minority language content.
Taiwan	Staff surge capacity for 24/7 monitoring; expand dialect coverage.	Institutionalize civic-platform compacts; standardize rumor metadata.	Integrate inforesponse drills with public health exercises.
The United States	Adopt minimal viable national indicators; depoliticize basic guidance via nonpartisan public institutions.	Create a transparency audit framework independent of platforms; resource-poor health departments; and shared analytics.	Rebuild trust via longitudinal community partnerships and primary health care (PHC) investments.
Brazil	Support state or municipal monitoring hubs; protect health workers from harassment.	Scale community-based correction relays beyond urban centers; fund Portuguese natural language processing for safety.	National transparency standards; sustained PHC-linked vaccine communication.
India	Scale rapid translation pipelines; strengthen tip lines; partner with radio or TV for corrections.	Privacy-preserving metadata collaboration; expand media literacy in rural schools.	Invest in multilingual artificial intelligence classifiers and community measurement panels.

## Research Agenda (Targeted)

The targeted research agenda consisted of the following:

- Effectiveness and cost-effectiveness: Comparing preventive literacy investments with reactive monitoring across baseline capacities.
- Causal pathways: Estimating how reductions in detection latency translate into behavior change and trust repair.
- Encrypted environments: Testing privacy-preserving analytics and community relay models without content access.
- Multilingual artificial intelligence: Training and evaluating safety models for underresourced languages, and audit parity.
- Participation and ethics: Measure impacts (benefits and harms) with governance by affected communities.

## Role of Digital Health Professionals and Multisector Partnerships

Health professionals, informaticians, and public health communicators occupy a connective role, translating complex scientific updates into accessible, culturally congruent narratives while flagging emergent misinformation trends to monitoring teams. Competency frameworks should incorporate skills in risk communication, data interpretation of infodemic metrics, and ethical engagement on social platforms. Partnerships with platform trust and safety teams can expedite escalation pathways for high-risk false therapeutic claims. Collaboration with educators and community health workers enhances reach into populations less accessible through digital channels alone. Professional bodies can issue guidance on balancing correction efforts with the avoidance of inadvertent amplification and provide psychosocial support structures addressing harassment risks documented in politicized environments.

## Ethical and Equity Considerations

Efforts to curtail harmful misinformation must guard against suppressing legitimate dissent or culturally rooted health practices. To ensure participatory governance in measurement, we recommend the establishment of community advisory boards that include representatives from marginalized groups such as indigenous communities, linguistic minorities, and civil society organizations at all stages of indicator selection, data collection, and evaluation. These boards should be empowered to co-design metrics, vet survey instruments for cultural relevance, and participate in interpreting results, thereby ensuring that measurement frameworks reflect local priorities and lived realities.

Contextual review panels should include indigenous knowledge holders and civil liberties advocates to vet proposed moderation criteria, ensuring that interventions respect local epistemologies and do not inadvertently delegitimize traditional health knowledge. Algorithmic fairness audits must go beyond technical assessment by involving speakers of underrepresented languages and dialects in user testing and error analysis to proactively identify and correct performance gaps that could reinforce linguistic or social inequities.

To mitigate risks associated with top-down interventions in low-capacity settings, external actors, especially those from high-income contexts, should prioritize local leadership and capacity transfer. This can be operationalized by requiring that all externally-funded infodemic management projects include a formal mechanism for shared decision-making with local institutions, transparent reporting of power dynamics, and a clear timeline for capacity handover. Where possible, interventions should be piloted in partnership with local organizations, with iterative feedback loops to ensure responsiveness to community concerns and evolving needs.

Data collaborations require principled minimization: only metrics essential to public health objectives should be shared, with clear and accessible public reporting of governance arrangements, data uses, and safeguards. Evaluation dissemination should include summaries in local languages and accessible formats (eg, audio and infographics) and should be presented in community forums to democratize oversight and invite critical feedback.

Finally, ongoing monitoring of both intended and unintended impacts, including surveillance, exclusion, or reputational harm, should be built into all infodemic interventions, with clear grievance and redress mechanisms available to affected communities.

## Limitations

This viewpoint is subject to several important limitations that warrant critical consideration. First, the comparative approach relies on case studies selected for their diversity and data availability, which introduces potential selection bias. Countries such as Brazil and India are highlighted due to their prominent infodemic challenges and the relative accessibility of public data, but this selection may overlook other contexts facing equally severe, yet less documented, infodemic impacts.

Second, the analysis is constrained by the limitations of available data, particularly in low-resource and marginalized settings. Much of the evidence cited is drawn from urban centers, platform-reported statistics, and civil society monitoring projects, which may underrepresent rural, tribal, and linguistically diverse communities. The lack of systematic, nationally representative measurement restricts the generalizability of findings and may obscure equity-sensitive impacts.

Third, the comparative framework risks overgeneralization by extrapolating lessons across heterogeneous sociopolitical and technological environments. Differences in governance structures, digital literacy, and health system capacity mean that interventions effective in one context may not translate directly to another. Furthermore, reliance on self-reported outcomes and platform transparency reports introduces potential reporting bias and limits the precision of key metrics such as detection latency and trust trajectories.

Finally, the challenge of standardizing infodemic management indicators across diverse settings remains unresolved. Variations in data infrastructure, language, and media ecosystems complicate efforts to benchmark progress and compare outcomes. Addressing these limitations will require ongoing investment in inclusive, participatory monitoring and the development of context-sensitive measurement tools.

## General Caveats When Drawing Lessons

Findings are illustrative and context-bound. Several indicators rely on urban-centric or platform-reported data. Where evidence is preliminary (eg, rumor half-life in Brazil and detection metrics on encrypted channels in India), treat claims as indicative rather than generalizable and flag evidentiary strength in-text. See monitoring and measurement constraints in Brazil and India, the World Health Organization’s guidance on social listening, and systematic review evidence [38,41,49,52-54].

## Conclusion

The infodemic equity gap conceptualized here reframes health misinformation not as an undifferentiated global wave but as a stratified challenge shaped by layered capacities whose uneven distribution yields predictable disparities in exposure, trust erosion, and behavioral misalignment. Comparative archetypes illustrate that resilience is constructed through coherent interaction of structural capacity, ecosystem governance, literacy and trust infrastructures, and adaptive intervention tooling. Measurement standardization and equity disaggregation are preconditions for accountable progress. Without deliberate investments that prioritize translation coverage, literacy institutionalization, transparent algorithmic governance, and participatory adaptive systems, existing asymmetries will widen under future health crises. Bridging this gap is both a digital health systems objective and a broader equity imperative, demanding sustained collaboration among public institutions, platforms, civic technologists, researchers, and communities. The framework and agenda advanced herein aim to catalyze that shift from episodic reaction to structured, equity-centered preparedness.

## References

[1] (2020). Managing the COVID-19 infodemic: call for action.

[2] Eysenbach G (2020). How to fight an infodemic: the four pillars of infodemic management. J Med Internet Res.

[3] Vosoughi S, Roy D, Aral S (2018). The spread of true and false news online. Science.

[4] Kotilainen S, Suoninen A, Arnala I (2021). Young people’s media literacy practices in Finland. J Media Lit Educ.

[5] Kupiainen R, De Abreu B, Mihailidis P, Lee A, Melki J (2017). International Handbook of Media Literacy Education.

[6] Resende G, Melo P, C. S. Reis J, Vasconcelos M, Almeida JM, Benevenuto F Analyzing textual (mis)information shared in whatsapp groups.

[7] Motta M, Stecula DA, Farhart C (2020). How right-leaning media coverage of COVID-19 facilitated the spread of misinformation in the early stages of the pandemic in the U.S. Can J Pol Sci.

[8] Jamieson KH, Albarracin D (2020). The relation between media consumption and misinformation at the outset of the SARS-CoV-2 pandemic in the US. Harv Kennedy Sch Misinformation Rev.

[9] Hallal PC, Victora CG (2021). Overcoming Brazil’s monumental COVID-19 failure: an urgent call to action. Nat Med.

[10] (2020). COVID-19 in Brazil: “So what?”. The Lancet.

[11] (2023). Measuring digital development: facts and figures 2023.

[12] (2024). The mobile economy 2024.

[13] Sørensen K, Pelikan JM, Röthlin F (2015). Health literacy in Europe: comparative results of the European health literacy survey (HLS-EU). Eur J Public Health.

[14] (2022). Regulation (EU) 2022/2065 of the European parliament and of the council on a single market for digital services (Digital Services Act). European Commission.

[15] (2021). Public health research agenda for managing infodemics.

[16] Banaji S, Bhat R (2020). WhatsApp vigilantes: an exploration of citizen reception and circulation of WhatsApp misinformation linked to mob violence in India. Glob Media Commun.

[17] Cheng HY, Li SY, Yang CH (2020). Initial rapid and proactive response for the COVID-19 outbreak - Taiwan’s experience. J Formos Med Assoc.

[18] Wang CJ, Ng CY, Brook RH (2020). Response to COVID-19 in Taiwan. JAMA.

[19] Santos M, Faure A (2018). Affordance is power: contradictions between communicational and technical dimensions of whatsapp’s end-to-end encryption. Social Media + Society.

[20] (2021). Standard Eurobarometer 94: media use in the European Union.

[21] Finnish Institute for Health and Welfare (2023). COVID-19 vaccination coverage in Finland: weekly report 2023.

[22] Sormunen M, Heikkilä V, Saaranen T, Kääriäinen M (2022). Teacher education for media literacy: a Finnish perspective. Nordic J Digital Lit.

[23] Chen CY, Zhang K, Zhang L (2021). Sharing health data and combating infodemics in Taiwan. Health Secur.

[24] Lin C, Braund WE, Auerbach J (2020). Policy decisions and use of information technology to fight COVID-19, Taiwan. Emerg Infect Dis.

[25] Allcott H, Gentzkow M, Yu C (2019). Trends in the diffusion of misinformation on social media. Res Polit.

[26] (2022). 'Humour over rumour': Taiwan’s messaging during COVID-19. Reuters.

[27] (2023). Taiwan is using humor as a tool against coronavirus hoaxes. UNMC Global Center for Health Security.

[28] Lin C, Braund WE, Auerbach J (2020). Policy decisions and use of IT to fight COVID-19, Taiwan. Emerg Infect Dis.

[29] Hsiao WWW, Lin JC, Fan CT, Chen SSS (2022). Precision health in Taiwan: a data-driven diagnostic platform for the future of disease prevention. Comput Struct Biotechnol J.

[30] Grossman G, Kim S, Rexer JM, Thirumurthy H (2020). Political partisanship influences behavioral responses to governors’ recommendations for COVID-19 prevention in the United States. Proc Natl Acad Sci USA.

[31] Muric G, Wu Y, Ferrara E (2021). COVID-19 vaccine hesitancy on social media: building a public dataset of user-generated content to identify key determinants. JMIR Public Health Surveill.

[32] Hernandez RG, Hagen L, Walker K, O’Leary H, Lengacher C (2021). The COVID-19 vaccine social media *infodemic* : healthcare providers’ missed dose in addressing misinformation and vaccine hesitancy. Human Vaccines & Immunotherapeutics.

[33] Wallace J, Goldsmith-Pinkham P, Schwartz JL (2023). Excess death rates for republican and democratic registered voters in Florida and Ohio during the COVID-19 pandemic. JAMA Intern Med.

[34] Guess AM, Lerner M, Lyons B (2020). A digital media literacy intervention increases discernment between mainstream and false news in the United States and India. Proc Natl Acad Sci U S A.

[35] Lhila A, Alghanem F (2023). Along party lines: Examining the gubernatorial party difference in COVID-19 mortality rates in U.S. counties. Prev Med Rep.

[36] (2025). Media literacy in education: policy brief. OSCE.

[37] Neely S, Witkowski K (2024). Social media authentication and users’ assessments of health information: random assignment survey experiment. JMIR Form Res.

[38] (2023). Misinformation monitoring reports [Web page in Portuguese]. Aos Fatos.

[39] Barberia LG, Gómez EJ (2020). Political and institutional perils of Brazil’s COVID-19 crisis. Lancet.

[40] Casanova Á, Gonzaga L (2022). Factors associated with COVID-19 vaccine hesitancy in Brazil. PLoS ONE.

[41] Rocha YM, de Moura GA, Desidério GA, de Oliveira CH, Lourenço FD, de Figueiredo Nicolete LD (2023). The impact of fake news on social media and its influence on health during the COVID-19 pandemic: a systematic review. J Public Health (Berl).

[42] Li SL, Prete CA, Zarebski AE (2024). The Brazilian COVID-19 vaccination campaign: a modelling analysis of sociodemographic factors on uptake. BMJ Open.

[43] Bastos LSL, Aguilar S, Rache B (2022). Primary healthcare protects vulnerable populations from inequity in COVID-19 vaccination: an ecological analysis of nationwide data from Brazil. The Lancet Regional Health - Americas.

[44] Boing AC, Boing AF, Borges ME, Rodrigues D de O, Barberia L, Subramanian S (2024). Spatial clusters and social inequities in COVID-19 vaccine coverage among children in Brazil. Ciênc saúde coletiva.

[45] Pescarini JM (2023). Vaccine coverage and effectiveness against laboratory-confirmed COVID-19 in Brazil. BMC Public Health.

[46] Endo PT, Santos GL, de Lima Xavier ME (2022). Illusion of truth: analysing and classifying COVID-19 fake news in Brazilian Portuguese language. BDCC.

[47] Azevedo NH (2025). Thematic trends in factchecking in Brazil’s COVID19 coverage. Annals of the Brazilian Academy of Sciences.

[48] (2022). Digital literacy initiatives: annual report 2022. Ministry of Electronics & Information Technology, Government of India.

[49] (2022). Annual report. https://www.altnews.in/annual-report-2022/.

[50] Badrinathan S (2021). Educative interventions to combat misinformation: evidence from a field experiment in India. Am Polit Sci Rev.

[51] Senjam SS, Manna S, Goel G (2024). Vaccination coverage against COVID-19 among rural population in Haryana, India: a cross-sectional study. PLoS One.

[52] Freitas Melo P Measuring message forwarding on WhatsApp.

[53] (2022). COVID-19 infodemic management: policy brief. WHO.

[54] (2025). Social listening in infodemic management for public health. WHO.

[55] (2022). 2022 strengthened code of practice on disinformation. European Commission.

[56] Pennycook G, Epstein Z, Mosleh M, Arechar AA, Eckles D, Rand DG (2022). Accuracy prompts are a replicable and generalizable way to reduce misinformation online. Nat Commun.

[57] Roozenbeek J, Linden S, Nygren T (2022). Psychological inoculation improves resilience against misinformation: evidence from a 15-language field. Sci Adv.

[58] Epstein Z, berinsky adam, Cole R, Gully A, Pennycook G, Rand DG Developing an accuracy-prompt toolkit to reduce COVID-19 misinformation online. PsyArXiv.

[59] Schlag G (2023). European Union’s regulating of social media: A discourse analysis of the Digital Services Act. PaG.

[60] Roozenbeek J, Linden S (2020). Global vaccination “Bad News” prebunking study. HKS Misinformation Review.

